# Incorporation of Concrete Polishing Waste as a Partial Substitute for Cement in Mortar

**DOI:** 10.3390/ma18030530

**Published:** 2025-01-24

**Authors:** Farjallah Alassaad, Houssam Affan, Bechara Haddad, Abdelrahman Mohamad, Nassim Sebaibi

**Affiliations:** 1 CMEG, Z.A. de Cardonville, Rue Compagnie D, 14740 Thue et Mue, France; 2 Groupe Vivialys, 37 Rue de Molsheim, 67000 Strasbourg, France; 3 Builders Lab, Builders Ecole d’ingénieurs, COMUE Normandie Université, 1 Rue Pierre et Marie Curie, 14610 Epron, France; 4 Vicat, 4 Rue Aristide Berges, 38080 L’Isle-d’Abeau, France

**Keywords:** concrete polishing waste, cement substitution, environmental sustainability, carbon footprint

## Abstract

This study examines concrete polishing waste (CFPW) potential as a partial cement substitute in mortar formulation. Concrete polishing waste, a by-product of the grinding and polishing of concrete surfaces, is mainly composed of fine particles of silica and calcium carbonate. The aim of the research was to assess this industry waste incorporation impact on various mortar properties. Four mixes containing different percentages of CFPW were tested for their physic-mechanical properties and environmental impact. The results show that increasing the CFPW percentage leads to higher porosity and shrinkage, as well as lower mechanical strength and density. However, a significant reduction in CO_2_ emissions was observed with CFPW incorporation (up to 29% reduction for 30% CPFW). Although CFPW use presents technical challenges, it offers an interesting opportunity to reduce mortars’ carbon footprint. The study concludes that moderate CFPW use can offer a balance between environmental benefits and performance, highlighting the need to optimize formulations to maximize benefits while minimizing compromises on mechanical properties.

## 1. Introduction

Concrete is the world’s most widely recognized building material, renowned across the globe for its adaptability, strength and durability. Its applications range as far afield as the foundations of high-rise buildings to sidewalks on commuter streets, making it an essential component of modern infrastructure. However, the pervasive material comes at a heavy environmental cost. Cement production, a prime ingredient in concrete, accounts for around 7% of global carbon dioxide (CO_2_) emissions [1]. The main reasons for this high level of emissions are the clinker production process, which requires a lot of energy, and the calcination of limestone, which releases CO_2_. Cement production has a considerable environmental impact. Raw materials, mainly limestone and clay, are extracted, crushed and ground into a fine powder. Cement powder is heated in a kiln to temperatures of up to 1450 °C to form clinker, which is subsequently ground with gypsum to produce cement [2,3]. Limestone calcination releases a significant amount of CO_2_ during this process. In addition, heating energy for the kiln adds a further contribution to greenhouse gas emissions. This double impact of raw material calcination and energy consumption renders cement production among highly carbon-intensive industrial processes.

Attempts aimed at reducing cement production’s carbon footprint involve developing alternative clinker compositions, improving energy efficiency and using alternative fuels. With growing climate change and environmental sustainability concerns, pressure is mounting on construction companies to reduce their carbon footprint. One successful strategy for reducing concrete’s environmental impact is by using supplementary cementitious materials (SCMs) [4,5,6,7,8,9,10]. Nevertheless, partial cement replacement with SCMs has proven highly effective as one of the most efficient strategies for reducing CO_2_ emissions. Not only do SCMs reduce the cement clinker content, but they also often make use of waste materials, thus contributing to waste reduction and resource efficiency.

SCMs refer to materials able to substitute partially for cement in concrete mixes, reducing both overall cement consumption and CO_2_ emissions. Avram et al. also revealed that the finest particles within sludge and slurry have a good binding ability to quartz particles, a fact that might be useful in preparing low-carbon-footprint mortars [11]. Such materials commonly include industrial by-products such as fly ash, silica fume and metakaolin. The incorporation of SCMs not only reduces concrete’s environmental footprint [12,13,14,15,16,17], but it can also improve its material properties, leading in many cases to enhanced durability and mechanical performance [18,19].

Polishing waste, a by-product of concrete polishing, is a promising alternative SCM. When polishing concrete, surfaces are sanded and polished to achieve a smooth, aesthetically finished appearance. During this process, fine particle waste, commonly known as concrete polishing waste (CFPW), is generated. The quantity of CFPW produced is important; for instance, it has been measured that polishing a concrete floor with a thickness of 1.5 mm results in around 9.5 kg of sludge per square meter, approximately 4 kg of which is CFPW powder [20]. If not properly treated, this waste creates environmental problems such as soil pollution and wasted resources [21,22].

Disposal methods for polishing waste are currently inefficient and unsustainable. The majority of this waste is discharged into landfills or stockpiled in open sites, adding to environmental degradation and representing a costly burden on industry. Considering its fine particle size combined with its presence of silica and calcium compounds, CFPW can be used as a cement replacement in concrete [20,23,24,25]. This potential use has a dual advantage: it solves the problem of waste disposal and reduces cement content in concrete, which in turn reduces CO_2_ emissions. Fine CFPW particles perform as a filler material, improving concrete matrix density and reducing voids. Improving the microstructure can result in increased concrete strength and durability. In addition, calcium oxide (CaO) in CFPW can contribute to and enhance the hydration process, improving concrete’s material properties even further [20].

On the other side, incorporating polishing waste into mortar not only offers the potential to improve concrete production’s environmental sustainability but at the same time also allies itself with broader goals of waste management and resource efficiency [26,27,28,29,30,31,32]. At a time when industries around the world are facing challenges in the disposal of industrial waste and need to reduce carbon emissions, novel approaches like using polishing waste in building materials might prove crucial in transitioning to more sustainable industrial practices.

Few studies have systematically assessed the impact of concrete polishing waste on the physical, mechanical and thermal properties of mortars while taking into account its environmental potential, leaving a gap in optimizing its incorporation as a partial cement substitute.

For this reason, the aim of this study is to systematically examine CFPW’s potential as a cement substitute in mortar. By examining various properties such as compressive strength, flexural strength, porosity, bulk density, water absorption, shrinkage and fire resistance, the study attempts to offer a comprehensive overview of the feasibility, as well as advantages, of using CFPW in building materials.

## 2. Materials and Characterization

The main materials used in this study are sand, gravel, cement, admixture and polishing waste. All these materials contribute significantly to the mortar’s properties and performance. This section presents a detailed characterization based on different methods.

### 2.1. Sand

Natural washed sand with a particle size distribution conforming to fine aggregate standards is used in this study. It is clean and free of impurities and has a density of 2630 kg/m^3^. The properties described in Table 1 include density, water absorption and other relevant characteristics.

### 2.2. Cement

Portland cement (CEM I 52.5R) is used as a binding agent. Characterized by its rapid hardening properties, this high-strength cement is frequently used in building construction. Cement’s chemical composition and physical properties are given in its technical data sheet, which includes data on fineness, density and compressive strength. Table 2 gives a detailed overview of cement’s chemical properties, while mechanical properties are shown in Table 3.

### 2.3. Polishing Waste

In this study, polishing wastes are derived from a concrete surface polishing process at an architectural concrete company (CMEG, Thue et Mue, France). Waste is generated during the sanding and polishing of concrete walls, using diamond abrasives to achieve a smooth finish. To avoid inhalation of fine particles, water is sprayed onto the surface during the polishing process, producing a sludge.

This sludge is collected and stored in airtight containers at room temperature. It is dried at 65 ± 5 °C to a constant mass. After drying, the sludge is manually ground and passed through an 80 µm sieve to obtain a fine powder suitable for use as SCM.

**Figure 1:**
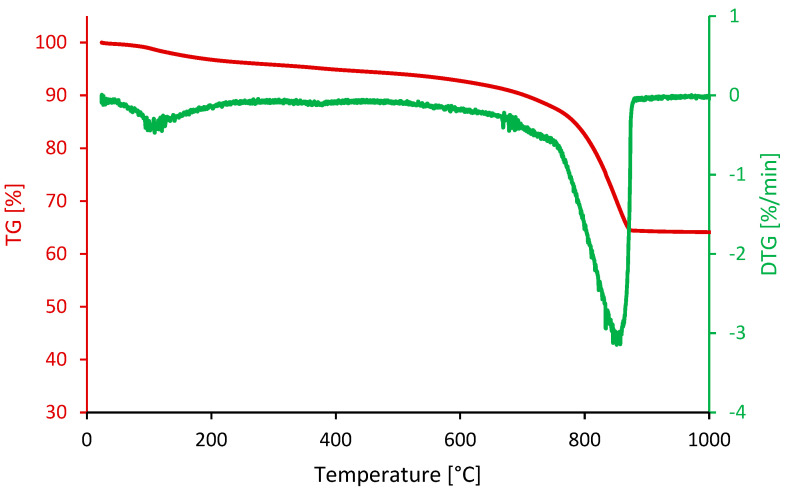
Thermogravimetric analyses of polishing waste.

Thermogravimetric analyses (TGA) are used for thermal stability and composition analysis of polishing waste. Measurement is performed in a nitrogen atmosphere, covering a temperature range from room temperature to 1000 °C in a Netzsch STA449 F3 Jupiter^®^ furnace (Selb, Germany). Thermogravimetric analysis of concrete polishing waste reveals several critical phases at different temperature ranges (see Figure 1). At temperatures below 120 °C, a decrease in mass is observed, corresponding to the evaporation of residual free water present in the samples. Between 120 and 600 °C, another significant phase is identified, indicating the release of bound water, probably related to the decomposition of calcium hydroxide (Ca(OH)_2_), with a peak in mass loss between 300 and 400 °C. Further mass loss is recorded between 600 and 1000 °C, suggesting the release of carbon dioxide (CO_2_) resulting from the decomposition of calcium carbonate (CaCO_3_), with a notable peak between 800 and 900 °C.

These observations are confirmed by the TGA curve, showing distinct peaks corresponding to each critical phase. In sum, TGA reveals the mass loss processes associated with the evaporation of free water, the release of bound water and the decomposition of calcium carbonate, providing crucial information for understanding the composition and thermal stability of concrete polishing waste. These data can guide appropriate treatment and recycling strategies for these materials.

Granulometric laser analysis is used to determine the particle size distribution of polishing waste. The results show that the particles are predominantly fine, with a large proportion below 80 µm in diameter.

**Figure 2:**
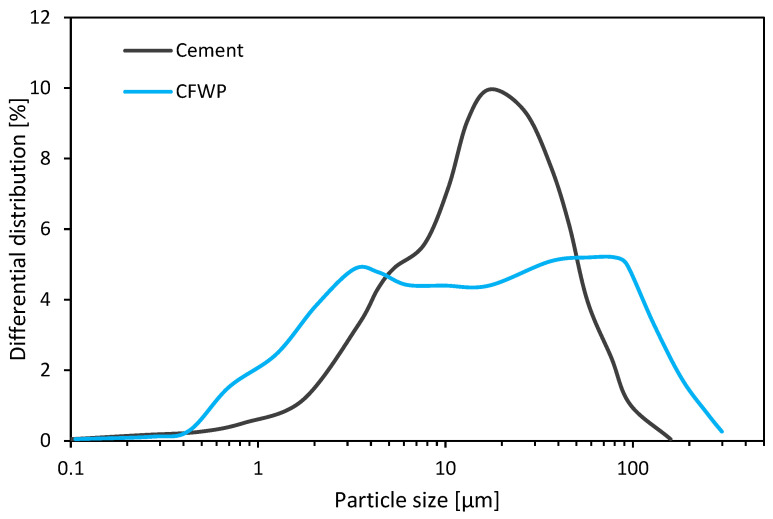
Particle size distribution of CFPW compared to cement.

The results in Figure 2 reveal a similar distribution to cement, with some differences. CFPW contains finer particles, probably originating from polished cement and filler. Meanwhile, coarser particles are present, originating from the fines in the sand used in the concrete. This particle distribution depends on the nature of the concrete produced. In general, sand particles are rounded, while CFPW quartz particles are probably elongated, with irregular edges due to polishing.

### 2.4. Method

Experiments were carried out to prepare and test mortar samples containing different percentages of polishing waste as a partial replacement for cement. In the following subsections, specific tests carried out to assess mortar performance are described.

### 2.5. Physical Properties

**Figure 3:**
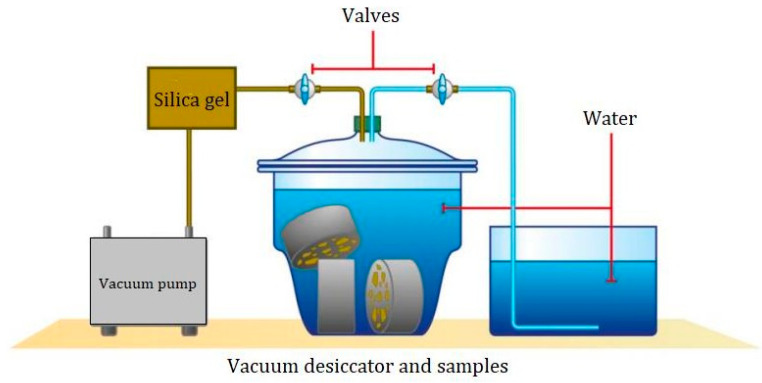
Illustration of water-accessible porosity process.

Mortar samples are tested for porosity and bulk density using the water displacement method. Oven-dried to a constant weight, samples are then immersed in water to determine water displacement (see Figure 3). Porosity is measured by dividing pore volume by total volume, and bulk density is calculated as dry sample mass divided by total volume.

Water-accessible porosity and bulk density were measured by hydrostatic weighing in accordance with standard NF P18-459 [33].

A further physical property term is water absorption. It is measured by immersing dried mortar specimens in water for 24 h. Samples are weighed before and after immersion to measure the amount of water absorbed. Water absorption rate is computed as the increase in mass due to water absorption, given as a percentage of sample dry weight.

### 2.6. Flexural Strength

**Figure 4:**
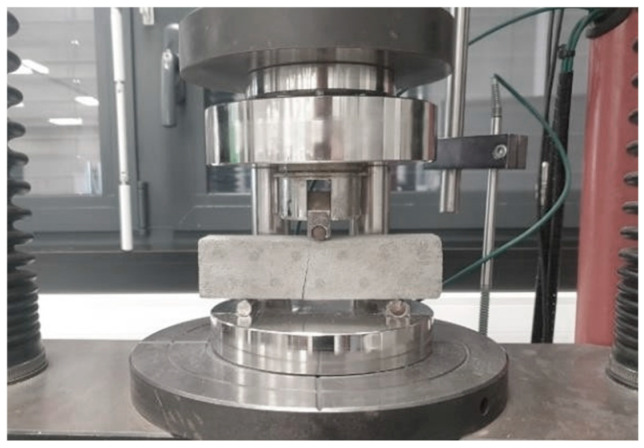
Flexural test device.

Mortar samples are tested for flexural strength using a three-point bending test in accordance with NF EN 196-1 [34] after 28 days of curing. Samples are prepared to standard dimensions (40 mm × 40 mm × 160 mm) and cured for several periods. The machine used is a 250 kN electromechanical press from IGM, equipped with a 50 kN load cell to improve result accuracy (see Figure 4). The standard requires a loading speed of 50 N/s. Three samples were tested per formulation.

### 2.7. Compressive Strength

**Figure 5:**
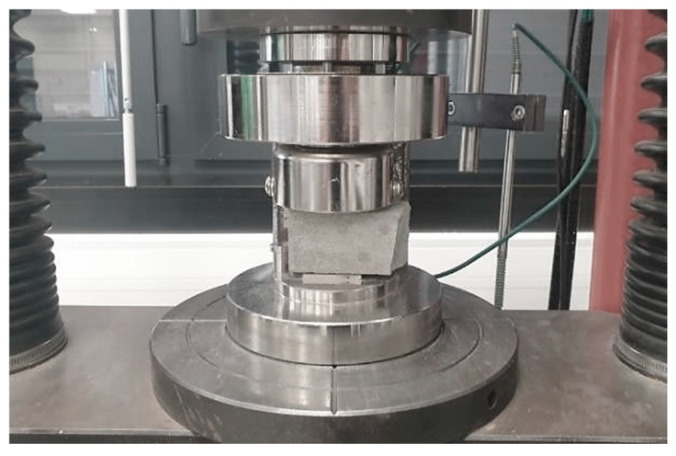
Compressive test device.

Following bending, compression testing was carried out on either half of the broken specimen (see Figure 5). The same press was used as for bending, except that the 50 kN load cell was replaced by one with a higher capacity (250 kN) with a cubic dimension (40 mm × 40 mm × 40 mm). Loading speed is 2.4 kN/s, in accordance with NF EN 196-1 [34].

Compressive strength is calculated on the basis of the maximum load applied, divided by the sample cross-sectional area.

### 2.8. Mortar Shrinkage

Shrinkage experiments are carried out to determine dimensional changes in mortar specimens over time. Mortar total shrinkage is assessed in accordance with standard NF P15-433 [35], which describes the method for measuring length variations in samples of hardened mortar. The test involves the use of prismatic specimens fitted with dowels to facilitate accurate measurement of length variation.

Samples (40 × 40 × 160 mm) are poured into molds and demolded after 24 h. Samples are then cured under controlled conditions (50% relative humidity and 20 °C temperature), and length changes are measured periodically using a length comparator. Total shrinkage is calculated in terms of length change compared to initial length.

### 2.9. Fire Resistance

Fire resistance was determined based on residual compressive strength following heating [36,37]. Each mixture was cast into 3 specimens and cured for 28 days. Following this, samples were kept in the muffle furnace at temperatures of 200 °C, 400 °C and 900 °C, respectively, for 1 h once the desired temperature had been reached. These temperatures are selected based on TGA results. After exposure to fire, specimens were removed from the oven and allowed to cool naturally. Once cooled, samples are visually inspected for cracks and spalling, and their flexural and compressive strength is measured.

### 2.10. Formulation

Four different mortar formulations are manufactured using various proportions of polishing waste: T (control, 0% waste), P10 (10% waste), P20 (20% waste) and P30 (30% waste). These percentages are selected to match standard cementitious additives such as limestone filler, metakaolin, etc. Mixing proportions are given in Table 4. Mixing consists of blending the dry ingredients, adding water and blending until a homogeneous mixture is obtained as follows:

- Add water first, then cement and CFPW; immediately afterward, start the mixer at low speed.
- After 30 s of mixing, add sand regularly for the next 30 s.
- Then, switch the mixer to high speed and continue mixing for a further 30 s.
- Stop the mixer for 1 min 30 s. During the first 15 s, scrape off any mortar adhering to the sides and bottom of the container, pushing it toward the middle of the container.
- Then, resume mixing at high speed for 60 s.
- The mortar is then poured into molds for hardening and subsequent testing.

## 3. Result and Discussion

### 3.1. Physical Properties

The results of physical property tests on mortars incorporating polishing waste are presented in Table 5. Adding these wastes increases porosity and decreases mortar density in proportion to their incorporation rate.

Porosity is 19.3% and density is 2008 kg/m^3^ for the reference mortar with no waste (Ref). With 10% waste (P10), porosity increases slightly to 19.5%, and density falls to 1973 kg/m^3^. This trend continues with higher waste contents, reaching a porosity of 22.6% and a density of 1942 kg/m^3^ for 30% waste (P30).

This increase in porosity and decrease in density can be explained by the intrinsically higher porosity of polishing waste compared to ordinary cement, as well as by the irregularity of its particles [38]. This creates a less compact arrangement and more voids in the cementitious matrix. However, too high a porosity can have a negative impact on other properties such as the mortar’s mechanical strength and durability.

The water absorption results for the different mortar formulations incorporating polishing waste are presented in Table 6.

The results show that the control formulation, without the addition of polishing waste, has a water absorption of 4.6%. This value serves as a benchmark against which other formulations can be compared. With 10% polishing waste, water absorption rises to 5.3%, indicating that even low incorporation of polishing waste influences water absorption. At 20% substitution, water absorption rises to 6.1%, showing an increasing trend as the percentage of polishing waste increases. The highest water absorption, 7.0%, is observed with 30% polishing waste, suggesting that water absorption continues to grow proportionally with increasing polishing waste content.

The water absorption of mortars increases significantly with the incorporation of polishing waste. Several factors may explain this trend. Concrete polishing waste is intrinsically more porous than traditional cement, which increases the mortar’s water absorption capacity. In addition, polishing waste particles are often irregular and can create voids in the cementitious matrix, facilitating water absorption. By replacing part of the cement with polishing waste, the amount of reactive binder material is reduced, which can affect the density and compactness of the mortar, thus increasing its water absorption.

### 3.2. Total Shrinkage

The results in Table 7 reveal a significant increase in total shrinkage when polishing waste is added to mortar formulations, with shrinkage increasing as waste content increases.

For the no-waste reference mortar (Ref), total shrinkage is 509 µm/m after 1 day, 603 µm/m after 3 days and 713 µm/m after 28 days and reaches 800 µm/m after 70 days.

By incorporating 10% waste (P10), shrinkage is already significantly higher: 833 µm/m at 1 day, 1081 µm/m at 3 days, 1147 µm/m at 28 days and 1306 µm/m at 70 days.

This trend becomes more pronounced with higher levels of waste. For P20 (20% waste), shrinkage peaks at 1491 µm/m after 70 days, around double the reference shrinkage. For P30 (30% waste), it reaches 16,013 µm/m at 70 days.

Thus, the incorporation of polishing waste, even at relatively low levels of 10%, results in much greater mortar shrinkage than conventional mortar. The phenomenon can be explained by the high fineness of waste particles hence drying shrinkage.

### 3.3. Flexural Strength

The flexural strength of mortars incorporating polishing waste at 14 and 28 days is presented in Table 8.

A progressive decrease in flexural strength is observed as the rate of cement substitution by polishing waste increases. This deterioration in flexural performance can be attributed to several factors. Firstly, polishing waste is composed mainly of fine particles of silica and calcium carbonate, which are much less reactive than anhydrous cement [39,40,41]. Their low hydraulic reactivity means that not enough hydration products can be developed to compensate for cement dilution and ensure adequate strength gain.

In addition, the irregular geometry of polishing waste particles creates localized stress concentrations. Their excessive fineness and higher intrinsic porosity also lead to lower compactness. This waste may also interfere with hydration kinetics and the development of optimum microstructure.

Thus, the low-reactivity nature of polishing waste, combined with unfavorable particle geometry and porosity effects, explains the significant degradation in flexural strength observed when this waste is incorporated in mortars in excessive quantities.

### 3.4. Compressive Strength

The compressive strength of the different mortar formulations incorporating polishing waste after 14 and 28 days of curing is shown in Table 9.

A significant decrease in compressive strength is observed as the rate of substitution of cement by polishing waste increases. This decrease is markedly accentuated for higher waste contents.

As with flexural strength, this deterioration in compressive performance can be attributed to the low reactivity of polishing waste. Indeed, these wastes are mainly composed of fine particles of silica and calcium carbonate, which develop few binding properties [39,40,41]. Their low hydraulic reactivity means that they cannot compensate for the dilution of reactive anhydrous cement by sufficient hydration products to ensure optimum development of mechanical strength.

In addition, other factors such as irregular particle geometry creating localized stress concentrations [42], excessive fineness leading to higher porosity and disturbances in cement hydration [43,44,45] also contribute to the observed reduction in compressive strengths, particularly at high substitution rates.

These findings concerning the decrease in compressive strength when increasing the substitution rate are in line with those of Wu et al. (2023) [39], who noted a comparable trend in their investigation of waste powders as cement mortar components.

### 3.5. Fire Resistance

The results in Table 10 for flexural strength after exposure to elevated temperatures show a gradual decrease in strength with increasing temperature. For the reference mortar (without CFPW), flexural strength after exposure to 200 °C, 400 °C and 900 °C is higher than for formulations containing CFPW. This is due to the chemical and physical nature of CFPWs, which have lower hydraulic reactivity and an irregular structure, adversely affecting the mortar’s internal cohesion after exposure to high temperatures.

The compressive strength in Table 11 of mortars after exposure to elevated temperatures follows a similar trend to that observed for flexural strength. Formulations containing CFPW show a significant reduction in compressive strength after exposure to 400 °C and 900 °C, compared with the reference formulation. This reduction is particularly marked for samples containing 20% and 30% CFPW. The low reactivity of CFPW and the presence of fine particles, which increase the porosity of the mortar, contribute to this loss of strength.

After exposure to fire, samples were visually inspected for cracks and flaking. Mortars containing higher percentages of CFPW (20% and 30%) showed an increased tendency of cracking and loss of surface cohesion, indicating a decrease in overall structural strength.

Incorporating CFPW into mortars offers significant environmental benefits, such as reduced CO_2_ emissions. However, from a fire resistance point of view, the results indicate that increasing CFPW content leads to a notable reduction in mechanical performance after exposure to elevated temperatures. This reduction in strength is due to the low reactivity of CFPW and the increased porosity of the mortar.

### 3.6. Embodied Carbon

The embodied carbon footprint represents the total greenhouse gas emissions associated with the production, transport, use and end of life of a material or product, expressed in CO_2_ equivalent. In this carbon impact calculation, several important assumptions were taken into account. Firstly, emission factors for raw materials are based on the ICE (Inventory of Carbon and Energy) database, with the exception of concrete polishing waste. The latter is assumed to have a similar environmental impact to screened gypsum, an assumption that may require further validation. The transport of constituents is uniformly estimated at 20 miles from the manufacturing unit for all formulations, which may not reflect the reality of all logistical situations. Concrete plant operations are considered to have a constant impact, regardless of formulation. Finally, an estimate of 1% mix waste was included in the calculations, representing an approximation of actual losses during the manufacturing process. These assumptions, while allowing a consistent comparison between different formulations, could be refined to reflect more accurately the specific production and logistical conditions of each manufacturing site.

Carbon impact analysis of the manufacture of mortars incorporating concrete polishing waste reveals a significant reduction in CO_2_ emissions compared with the reference mortar. Using the ICE database [46] for raw material emission factors, the study evaluates four formulations: Ref (no-waste reference), P10 (10% waste), P20 (20% waste) and P30 (30% waste). The raw material database embodied carbon is presented in Table 12.

**Figure 6:**
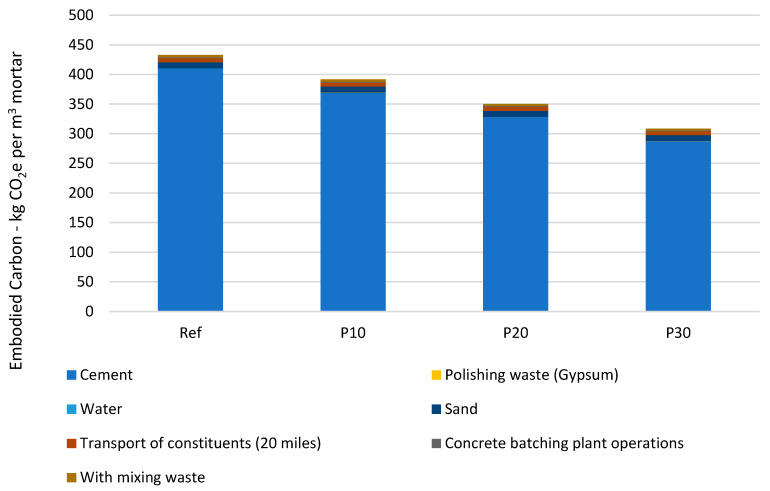
Embodied carbon of mortar incorporating polishing waste.

The results in Figure 6 show a gradual reduction in total emissions, from 433 kg CO_2_e/m^3^ for Ref to 309 kg CO_2_e/m^3^ for P30, a reduction of 28.6%. This is mainly attributable to the reduction in cement content, the main contributor to emissions, from 410.4 kg CO_2_e/m^3^ for Ref to 287.3 kg CO_2_e/m^3^ for P30.

Polishing waste, considered to have a similar impact to screened gypsum, makes a minimal contribution to emissions (0.1 to 0.3 kg CO_2_e/m^3^), underlining the environmental benefits of recycling it. The transport of components, assumed to be 20 miles from the manufacturing plant, has a relatively constant impact (around 6.5 kg CO_2_e/m^3^) between formulations. Other components such as sand (10.1 kg CO_2_e/m^3^), water and plant operations have a lesser but constant impact.

This study demonstrates the significant potential for reducing the carbon footprint of mortars by incorporating concrete polishing waste, with a reduction of up to almost a third in emissions for the P30 formulation. However, it is crucial to consider these results alongside the mechanical and durability performance of mortars, which may be affected by the incorporation of these wastes, in order to optimize the trade-off between environmental benefits and technical properties.

## 4. Discussion

The integration of concrete polishing waste into mortar formulations showed varied impacts on their physical, mechanical and thermal properties, revealing both technical opportunities and limitations.

In terms of physical properties, the results show an increase in porosity and a decrease in mortar density as a function of the rate of cement substitution by CFPW. These changes are linked to the inherently porous nature of CFPW and their irregular geometry, which reduces the compactness of the cementitious matrix. This increase in porosity is also accompanied by increased water absorption, which can affect mortar durability [39].

The mechanical performance of mortars, as measured by compressive and flexural strength, shows a gradual decline with increasing levels of CFPW. This loss of strength is due to the low hydraulic reactivity of CFPW, mainly composed of silica and calcium carbonate, which do not generate sufficient hydration products to compensate for the reduction in active cement in the mix [41]. Furthermore, the irregular shape of CFPW particles seems to exacerbate these effects, creating stress concentrations in the matrix.

In terms of shrinkage, mortars incorporating CFPW show a marked increase in total shrinkage compared with reference mortars. This trend is linked to the high fineness of CFPW particles, which influences the microstructure of the matrix and contributes to increased deformation over time. These dimensional variations can compromise the integrity of structures in demanding environments [18].

The thermal performance of mortars containing CFPW is also affected. After exposure to high temperatures (400 °C and 900 °C), a significant reduction in mechanical strength was observed. This fragility is due to the thermal decomposition of the carbonates present in CFPW, resulting in a loss of internal cohesion and an increase in mortar porosity [39].

Finally, in environmental terms, the results show that incorporating CFPW significantly reduces the carbon footprint of mortars, achieving a reduction of almost 29% for a 30% substitution. This reduction is mainly due to the reduction in the quantity of cement, which is the main contributor to CO_2_ emissions in mortars [20,32].

## 5. Conclusions

This study focused on the evaluation of concrete polishing waste as a partial substitute for cement in mortar formulations. CFPW, derived from the grinding and polishing of concrete surfaces, is mainly composed of fine particles of silica and calcium carbonate. The aim was to analyze the impact of CFPW incorporation on various mortar properties, such as mechanical strength, porosity, density, water absorption, shrinkage and fire resistance. Four mixes with different percentages of CFPW (0%, 10%, 20%, 30%) were tested for their physico-mechanical properties and environmental impact.

In environmental terms, the addition of CFPW leads to a significant reduction in CO_2_ emissions, with a reduction of almost 29% for a 30% substitution rate. This trend underlines the role of CFPW in reducing carbon footprints, particularly in a context where climate concerns are crucial.

From a physical point of view, a progressive increase in porosity (+17% to 30% CFPW) and water absorption was observed, accompanied by a decrease in density. These structural changes are attributed to the fineness and intrinsic porosity of CFPW particles.

In mechanical terms, compressive and flexural performance progressively decline with increasing CFPW. For example, compressive strength drops from 51.1 MPa for the control mortar to 23.7 MPa for a 30% substitution, representing a loss of almost 54%. A similar trend is observed for flexural strength, decreasing from 6.7 MPa to 4.0 MPa. These decreases are linked to the low hydraulic reactivity and structure of CFPW, which impact the cohesion of the cementitious matrix.

Finally, thermal performance shows marked degradation after exposure to high temperatures (400 °C and 900 °C), with a significant reduction in mechanical strength. These results point to structural embrittlement due to the decomposition of the carbonates present in CFPW.

In conclusion, although CFPW represents a sustainable option for recycling concrete waste, further research is needed to improve mortar formulations to meet mechanical performance and fire resistance requirements. Moderate use of CFPW balances environmental benefits with necessary performance, highlighting the need to optimize formulations to maximize benefits while minimizing compromises on mechanical properties.

## Figures and Tables

**Table 1:** Sand physical properties.

Properties	Washed 0/4 Sand
Density	2630 kg/m^3^
Water absorption	0.1%
Sand equivalent	81.3
Fineness modulus	2.3

**Table 2:** Cement chemical characteristics.

Chemical Characteristics [%]	CEM I 52.5 R–SR5 CE
SO_3_	2.1
MgO	0.6
Na_2_O	<0.3
Cl^−^	<0.04
Fire loss	1.1

**Table 3:** Cement mechanical properties.

Compressive Strength [MPa]	CEM I 52.5 N CE CP2 NF
1d	21–27
2d	40–48
7d	53–65
28d	66–76

**Table 4:** Mortar formulations.

Formulation [kg/m^3^]				
Component	Ref	P10	P20	P30
Cement	450	405	360	315
Polishing waste	0	45	90	135
Sand	1350	1350	1350	1350
Water	225	225	225	225

**Table 5:** Mortar physical properties.

Formulation	Porosity [%]	Density [kg/m^3^]
Ref	19.3 ± 0.9	2008 ± 10
P10	19.5 ± 0.9	1973 ± 9
P20	19.7 ± 1.0	1953 ± 15
P30	22.6 ± 0.8	1942 ± 14

**Table 6:** Water absorption of mortars incorporating washing fines.

Formulation	Water Absorption [%]
Ref	4.6 ± 0.2
P10	5.3 ± 0.2
P20	6.1 ± 0.2
P30	7.0 ± 0.2

**Table 7:** Total shrinkage of mortar incorporating polishing waste.

Formulation	Total Shrinkage [µm/m]			
	1 Day	3 Days	28 Days	70 Days
Ref	509 ± 5	603 ± 8	713 ± 15	800 ± 10
P10	833 ± 9	1081 ± 15	1147 ± 13	1306 ± 13
P20	713 ± 10	1153 ± 13	1297 ± 14	1437 ± 11
P30	800 ± 9	1288 ± 10	1491 ± 16	1613 ± 8

**Table 8:** Flexural strength of mortar incorporating polishing waste.

Formulation	Flexural Strength [MPa]	
	14 Days	28 Days
Ref	6.4 ± 0.4	6.7 ± 0.2
P10	5.5 ± 0.2	5.6 ± 0.2
P20	4.8 ± 0.3	5.0 ± 0.3
P30	3.7 ± 0.3	4.0 ± 0.2

**Table 9:** Compressive strength of mortar incorporating polishing waste.

Formulation	Compressive Strength [MPa]	
	14 Days	28 Days
Ref	47 ± 1.8	51.1 ± 1.2
P10	42.2 ± 1.2	44.1 ± 1.0
P20	33.7 ± 1.4	35.2 ± 1.1
P30	21.1 ± 1.3	23.7 ± 1.4

**Table 10:** Flexural strength of mortar after exposure to fire.

Formulation	Flexural Strength [MPa]		
	200 °C	400 °C	900 °C
Ref	5.8 ± 0.4	4.8 ± 0.3	0.6 ± 0.2
P10	5.7 ± 0.3	3.5 ± 0.2	0.5 ± 0.2
P20	4.1 ± 0.3	3.5 ± 0.1	0.4 ± 0.1
P30	4.0 ± 0.2	2.0 ± 0.2	0.3 ± 0.2

**Table 11:** Compressive strength of mortar after exposure to fire.

Formulation	Compressive Strength [MPa]		
	200 °C	400 °C	900 °C
Ref	27.7 ± 1.2	25.9 ± 1.5	4.4 ± 0.8
P10	21.0 ± 1.2	20.6 ± 1.8	4.1 ± 0.3
P20	17.7 ± 1.0	16.9 ± 1.2	3.4 ± 0.3
P30	13.5 ± 0.8	13.1 ± 0.6	2.7 ± 0.3

**Table 12:** Embodied carbon based on ICE database [46].

Material	Cement	Polishing Waste (Gypsum)	Water	Sand	Transport of Constituents (20 Miles)	Concrete Batching Plant Operations	With Mixing Waste
Embodied Carbon Factor—kg CO_2_e/kg	0.912	0.002536	0.000344	0.00747	0.00332626	0.004991	1.0%

## Data Availability

The raw data supporting the conclusions of this article will be made available by the authors on request due to confidentiality.

## Abbreviations

The following abbreviations are used in this manuscript:

CFPW	Concrete polishing waste or concrete fine polishing waste
SCMs	Supplementary cementitious materials
TGA	Thermogravimetric analyses
ICE	Inventory of Carbon and Energy
Ref	Reference mortar
P#	Mortar with # % CFPW as cement replacement
